# Associations Between Supporting Factors and Healthcare Utilization in People with Dementia: Data from the DelpHi Study

**DOI:** 10.1002/alz70858_102801

**Published:** 2025-12-26

**Authors:** Nadia Blecha, Iris Blotenberg, Lina Charlotte Jeran, Bernhard Michalowsky, Wolfgang Hoffmann, Jochen René Thyrian

**Affiliations:** ^1^ Deutsches Zentrum für Neurodegenerative Erkrankungen e. V. (DZNE), site Rostock / Greifswald, Greifswald, Mecklenburg‐Vorpommern, Germany

## Abstract

**Background:**

This study examined the relationship between healthcare utilization and support related factors, such as social support and marital status, in patients with dementia (PwD) over 5 years.

**Methods:**

We analysed data from 498 participants in the GP‐based randomized controlled intervention trial DelpHi‐MV (dementia: life‐ and person‐centred help in Mecklenburg‐Western Pomerania, Germany). Negative binomial regression was used to model GP and internist visits, while zero‐inflated negative binomial regression analysed dentist and neurologist/psychiatrist visits, with predictors including social support and sociodemographic variables.

**Results:**

Participants had a mean age of 80.1 years (SD = 5.45), with 59.6% female and 43.7% married. Emotional support (β = 0.23, SE = 0.04, *p* < 0.0001), social integration (β = ‐0.10, SE = 0.03, *p* = 0.003), and marital status (divorcés: β = 0.24, SE = 0.07, *p* < 0.0001; widowers: β = 0.08, SE = 0.04, *p* = 0.03) were associated with GP visits. Practical support was positively associated with dentist visits (b = 0.27, SE = 0.06, *p* < 0.0001). Social integration (b = 0.21, SE = 0.08, *p* = 0.03) and marital status (singles: b = 1.42, SE = 0.24, *p* < 0.0001) emerged as significant predictors for neurologist/psychiatrist visits.

**Conclusion:**

Our findings highlight the importance of social support, including emotional and practical support, social integration, and marital status, as key predictors of healthcare utilization in PwD. These results underscore the need for tailored interventions to address social and demographic disparities in dementia care.

